# Personal

**Published:** 1900-12

**Authors:** 


### PERSONAL.

Dr. E. L. Shurly, of Detroit, Mich., visited Buffalo, Monday, November 19, 1900, and participated in the proceedings of the Buffalo Academy of Medicine (laryngological section) in which there was a discussion on tuberculosis. Previous to the meeting Dr. Frank Whitehill Hinkel, chairman of the section, gave a dinner at the University Club, where Dr. Shurly, as the guest of honor, met a number of his old friends and some new ones as well. Among the guests were: Drs. Henry R. Hopkins, Charles G. Stockton, D. W. Harrington, Herman Mynter, F. W. Abbott, DeLancey Rochester, John H. Pryor, H. R. Gaylord and William Warren Potter.

Dr. H. A. Foster, of St. John's Place, accompanied by Mrs. Foster, has gone to Santa Paula, Cal., to spend the winter. Dr. Foster has recovered from a recent illness and avails himself of the mellow climate of Southern California to establish permanent recovery.

Dr. Lucien Howe, of Buffalo, was elected president of the Medical Association of Central New York during its recent annual meeting held at Rochester. Dr. Howe will preside at the next meeting, to be held in this city on the last Tuesday of September, 1901.

Dr. Martin J. Downey, of Buffalo, U. of B., '99, for more than a year connected with the staffs of the Sisters and Emergency Hospitals, has left the latter institution and will establish an office in Seneca Street about December 1st.

Dr. W. H. Heath and Dr. Nelson W. Wilson, of Buffalo, have removed their offices from No. 267 Franklin Street to No. 453 Niagara Street, corner of Hudson. Telephone Tupper 507. Consultation hours: 2-4, and after 7 p.m.

Dr. N. G. Russell, of Buffalo, lately appointed district physician by Health Commissioner Wende, has established his office at 251 Jefferson Street, corner of Clinton. Hours: 12 to 1 p.m. Telephone, Howard 364.

Dr. William B. May, of Buffalo, has removed from 658 Clinton Street to 251 Jefferson Street, corner of Clinton. Hours: 2-3 and 7-8 p.m. Telephone, Howard 364.

Dr. Julien A. Riester, U. of B., '99, has finished his term as resident physician at the Buffalo General Hospital and will make his home in Buffalo.

---

Dr. A. Walter Suiter, of Herkimer, N.Y., recently paid a visit to Buffalo as the guest of Dr. Ernest Wende and Dr. William Warren Potter.

---

Dr. J. M. O'Neill, of Buffalo, has removed from 852 Seneca Street to 536 Swan Street. Hours: 1-3 and 7-8 p.m. Telephone, Howard 212.

---

Dr. W. J. Deane, of Buffalo, has removed from 134 E. Ferry to 926 W. Ferry Street. Hours: 8-10 a.m.; 1-3 and 7-9 p.m.

---

Dr. Alton L. Smiley, of Buffalo, has been appointed a medical interne at the Buffalo State Hospital.

---

Dr. William Stanton, U. of B. '89, of Varysburg, N.Y., has removed to Webster, N.Y.

---

Dr. A. L. Benedict, of Buffalo, announces the removal of his office from 174 Franklin Street to 995 Main Street. Office hours and limitation of practice to diseases of the digestive organs, will remain as before. Telephone, Tupper 670.

